# Outcome predictors of uncomplicated sepsis

**DOI:** 10.1186/1865-1380-6-9

**Published:** 2013-04-08

**Authors:** Ewoud ter Avest, Maarten de Jong, Ineke Brűmmer, Götz JK Wietasch, Jan C ter Maaten

**Affiliations:** 1 Department of Emergency Medicine, Medical Center Leeuwarden, Henry Dunantweg 2, Leeuwarden, 8934 AD, The Netherlands; 2 Department of Emergency Medicine, University Medical Center Groningen, Groningen, The Netherlands; 3 Department of Anaesthesiology, University Medical Center Groningen, Groningen, The Netherlands

**Keywords:** Sepsis, Uncomplicated, Emergency department, Outcome

## Abstract

**Background:**

The development of sepsis risk prediction models and treatment guidelines has largely been based on patients presenting in the emergency department (ED) with severe sepsis or septic shock. Therefore, in this study we investigated which patient characteristics might identify patients with an adverse outcome in a heterogeneous group of patients presenting with uncomplicated sepsis to the emergency department (ED).

**Findings:**

We performed a retrospective cohort analysis of all ED patients presenting with uncomplicated sepsis in a large teaching hospital during a 3-month period. During this period, 70 patients fulfilled the criteria of uncomplicated sepsis. Eight died in the hospital. Non-survivors were characterized by a higher abbreviated Mortality in Emergency Department Sepsis (MEDS) score (7.2 ± 3.4 vs. 4.8 ± 2.9, *p* = 0.03) and a lower Hb (6.6 ± 1.2 vs. 7.7 ± 1.4, *p* = 0.03), and they used beta-blockers more often (75% vs. 19%, *p <* 0.01).

**Conclusions:**

Non-survivors of uncomplicated sepsis had on average a higher abbreviated MEDS score, a lower hemoglobin (Hb) and more often used β-blockers compared to survivors. Early identification of these factors might contribute to optimization of sepsis treatment for this patient category and thereby prevent disease progression to severe sepsis or septic shock.

## Introduction

Sepsis remains an ongoing challenge in medicine. Mortality rates depend on the sepsis stage and co-morbidity and range from 4.1% in patients with uncomplicated sepsis to as high as 50% in patients with septic shock [1]. Although patients with severe sepsis or septic shock have a higher a-priori chance of having an unfavorable outcome than patients with uncomplicated sepsis, the latter group is responsible for the majority of sepsis hospitalizations in developed countries [2]. Unfortunately, however, research over the past years has mainly been focused on the early identification and treatment of patients with advanced stage sepsis: Risk prediction models and especially treatment guidelines for sepsis at the ED are largely derived from patients presenting with severe sepsis or septic shock, and it remains unclear whether the same risk prediction models are applicable for patients presenting to the ED with uncomplicated sepsis [3-5]. Therefore, the primary objective of this study was to investigate which patient characteristics are related to outcome in a heterogeneous group of patients presenting with uncomplicated sepsis to the ED.

## Methods

### Study design and inclusion criteria

We performed a retrospective cohort analysis of all adult emergency department (ED) patients (>18 years) who presented to the ED of the University Hospital Groningen with uncomplicated sepsis during a 3-month period (1 September 2010 until 30 November 2010). During this period, a quality improvement initiative had been carried out to improve early sepsis recognition in patients with sepsis at the ED. According to this initiative, for all patients visiting the ED during the study period with sepsis (defined as the presence of two or more SIRS criteria in combination with the suspicion of a *new* infection), temperature, heart rate, respiration rate, blood pressure and oxygen saturation were recorded at presentation by a trained triage nurse. Ordering of (additional) diagnostic tests and initiation of therapy were both left completely to the discretion of the attending physician.

In the present retrospective cohort analysis, only patients with non-severe (i.e., uncomplicated) sepsis were enrolled. Uncomplicated sepsis was defined retrospectively by one of the authors (MdJ) as sepsis in the absence of signs of organ dysfunction at a site remote from the site of the infection or sepsis with signs of hypotension or hypoperfusion. Subjects were excluded when one or more of the following criteria were met for not to being considered to have a chronic condition: systolic blood pressure <90 mmHg, or a MAP<65 mmHg, creatinine >177 umol.l^-1^, bilirubin >34 mmol.l^-1^, platelet count <100×10^9^.l^-1^, coagulopathy with INR>1.5 or aPTT > 60 s, or a lactate level of > 2 mmol.l^-1^. A limited life expectancy as a result of underlying disease was not an exclusion criterion in the present study.

### Data collection

Vital signs, biochemical test results, ED treatment characteristics and final outcome were retrieved from the electronic patient files. Abbreviated Mortality in Emergency Department sepsis (MEDS) scores [3] without neutrophil bands [6] (since these are not routinely measured in many Dutch hospitals) were calculated retrospectively from eight independent predictors of mortality, resulting in a maximum score of 24.

### Primary data analysis

Differences between survivors and non-survivors regarding baseline characteristics were tested for statistical significance by using the two-tailed Fisher’s exact test for dichotomous variables and one-way ANOVA for continuous variables, with age, gender, time-to-antibiotics and fluid administration (all variables) and MEDS score (treatment variables) as covariates. It was estimated that with our study population of 70 subjects, a Spearman’s correlation coefficient (*r*) of 0.3 could be detected with a power of 80% and an alpha error of 0.05.

### Ethics

As our study only involved retrospective evaluation of routinely recorded patient data, this type of study was determined to be exempt research by our local institutional review board.

## Findings

During the 3-month period, 8,146 patients visited the ED of the University Medical Center Groningen. During this period, 101 patients were diagnosed with sepsis, of which 31 had a severe sepsis or septic shock, and 70 had an uncomplicated sepsis. Further results refer to this latter group.

### Clinical and biochemical characteristics at presentation

Eight patients died in-hospital after presentation to the ED, on average 11 days after presentation (range 4–21). Table 1 shows the clinical and biochemical characteristics at presentation of survivors and non-survivors. The non-survivors were on average 14 years older than the survivors. Although they more often had an underlying medical condition predisposing for sepsis, only one of the non-survivors had a terminal illness (metastatic malignancy) with a life expectancy <1 month at the time of presentation (Tables 1 and 2). Non-survivors more often used beta-blockers compared to survivors, while other vasoactive drug use and immunosuppressive drug use were not significantly different between both groups. Lower respiratory tract infections were the prevailing source of sepsis in both survivors (56%) and non-survivors (75%, *p* = 0.77). Hemoglobin concentration was the only biochemical parameter that differed significantly between survivors and non-survivors, even after correction for the age difference between both groups. As expected, the mean MEDS score was significantly higher in patients with an adverse outcome compared to survivors. None of the subjects had a MEDS score ≥12 (high-risk group).

**Table 1:** Clinical and biochemical characteristics of survivors and non-survivors of uncomplicated sepsis at the emergency department

	***n***	**Survivors (*n*= 62)**	**Non-survivors (*n*= 8)**	***p***
		***n*[%]**	***n*[%]**	
**Demographics and history**				
Gender (M/F)	70	35/27	4/4	0.51
Age (years)	70	57 ± 19	71 ± 9	0.04
Underlying predisposing condition for adverse course of sepsis*	70	39 [63%]	8 [100%]	0.42
Immunocompromised state**	70	22	4	0.73
Nursing home resident	70	1 [2%]	1 [12%]	0.24
Terminal illness	70	1 [12%]	1 [12%]	0.24
**Relevant medication use**				
Immunosuppressive drugs (*n*)	70	19 [31%]	1 [12%]	0.68
Maintenance antibiotics (*n*)	70	5 [8%]	1 [12%]	0.54
β-blockers (*n*)	70	12 [19%]	6 [75%]	<0.01
Other vasoactive medications	70	18 [29%]	3 [38%]	0.71
Statins	70	15	1	1.00
**Site of infection**				
Lower respiratory tract infection	70	35	6	0.77
Urinary tract infection	70	10	2	0.64
Other	70	17	0	0.35
**Vital signs**				
Altered mental state (GCS <15)	70	0	1	0.13
Heart rate (bpm)	70	109 ± 18	101 ± 19	0.34
Respiration rate (rpm)	70	24 ± 6	22 ± 5	0.14
Temperature (°C)	70	38.3 ± 1.0	37.8 ± 1.6	0.32
SBP (mmHg)	70	131 ± 26	132 ± 30	0.62
DBP (mmHg)				
MAP (mmHg)	70	91 ± 16	95 ± 20	0.84
Oxygen saturation (%)	70	94 ± 4	95 ± 3	0.58
**Laboratory values**				
Hb (mmol.l^-1^)	70	7.7 ± 1.4	6.6 ± 1.2	0.03
Ht (%)	70	36 ± 0.10	35 ± 0.09	0.94
Platelet count (×10^9^.l^-1^)	69	251 ± 160	243 ± 179	0.77
White blood cell count (×10^9^.l^-1^)	70	13.0 ± 6.2	8.5 ± 4.7	0.06
CRP (mg.l^-1^)	70	110 ± 98	113 ± 90	0.90
Creatinine (umol.l^-1^)	70	83 ± 49	79 ± 20	0.58
BUN (mmol.l^-1^)	70	6.3 ± 3.3	9.2 ± 3.6	0.14
Bilirubin (mmol.l^-1^)	49	18 ± 21	21 ± 12	0.93
Glucose (mmol.l^-1^)	61	7.2 ± 2.5	6.3 ± 1.0	0.23
Arterial lactate (mmol.l^-1^)	45	1.1 ± 0.5	1.3 ± 0.2	0.57
**Treatment characteristics**				
Time to antibiotics (min)	47	88 ± 54	80 ± 51	0.85
Fluid administration first 120 min (ml)	62	688 ± 586	625 ± 627	0.89
**Abbrev. MEDS score**	70	4.8 ± 2.9	7.2 ± 3.4	0.03

Data are presented as mean ± SD. GCS, Glasgow coma scale; SBP, systolic blood pressure; MAP, mean arterial pressure; Hb, hemoglobin; CRP, C-reactive protein; BUN, blood urea nitrogen.

*See Table 2.

**Immunocompromised state was considered present if a patient had any of the following: HIV or AIDS, leukemia, any malignancy, any history of chemotherapy, neutropenic fever, non-Hodgkin’s lymphoma, transplanted organ, or current use of steroid or other immunosuppressive drugs.

**Table 2:** Co-morbidity of survivors and non-survivors of uncomplicated sepsis at the emergency department

	**Survivors (*n*= 62)**	**Non-survivors (*n*= 8)**	***p***
	***n*[%]**	***n*[%]**	
Active malignancy	7	4	0.02
-With metastasis	1	1	0.22
Status after organ transplantation	7	1	0.64
CAD or urinary tract abnormalities	6	1	0.59
Structural bile-duct abnormalities	5	0	0.54
Heart failure	11	3	0.19
Diabetes mellitus	10	1	0.63
Chronic obstructive pulmonary disease	12	3	0.27
Cystic fibrosis	1	0	0.89
Wegener’s granulomatosis	1	0	0.89

### Treatment characteristics

Table 1 shows the treatment characteristics for both survivors and non-survivors. Mean time from presentation at the ED to the administration of the first gift of antibiotics was 87 min (range 15–215 minutes). The average amount of intravenous fluids administered was 680 ± 584 ml in the first 120 min. No significant differences regarding time to antibiotics or the amount of fluid administration during the first 120 min at the ED were present between survivors and non-survivors, even after correction for the difference in MEDS score between both groups (to correct for possible treatment differences resulting from differences in disease severity).

### Association of patient characteristics with outcome

Of all clinical and biochemical variables at presentation, only beta-blocker use was correlated to outcome with a correlation coefficient (*r*) larger than the minimum *r* of 0.3 that could be detected with our study population (*r* = −0.40, *p* < 0.001).

## Discussion

In our study, we found a mortality rate of 11%, which is higher than several previous studies reported [3,5]. It is unlikely that the higher mortality rate in our study is the result of the inclusion of many subjects suffering from a terminal illness: Only 2 out of 70 subject had a terminal illness and a resulting do-not-resuscitate status. All other subjects received optimal care, including referral to the ICU ward. An alternative explanation however might be that 56 out of 70 subjects in our study had co-morbidity potentially predisposing them to an adverse course of their sepsis, and 26 out of 70 subjects (37%) were considered immunocompromised.

Previous studies identified various patient characteristics related to final outcome in patients with severe sepsis, and multiple risk prediction models have been developed to adequately risk stratify patients based on combinations of these characteristics [7]. Most commonly used, and the only model created to aid clinical decision making in the ED, is the MEDS score [3]. In our study we calculated abbreviated MEDS scores (without neutrophil bands, reflecting common practice in the Netherlands) as a measure of sepsis severity [6]. Even though the MEDS score was <12 for all subjects included in our study, we found that the average MEDS score was significantly higher in subjects with an adverse outcome [7,8], which was mainly a reflection of the difference in age between both groups. In our study, however, the non-survivors differed from the survivors also in respect to various parameters not included in the MEDS score. First, subjects with an adverse outcome invariably had an underlying predisposing medical condition for their sepsis. Second, they more often used beta-blockers compared to survivors. This finding is in line with several experimental animal studies that do report an increased overall sepsis mortality in beta-blocker subgroups [9] despite the well-known positive effects of beta-blockers on glucose modulation, the host immune response and sepsis-related cardiac dysfunction [10]. This might be explained by the masking effects of beta-blocker use on tachycardia, resulting in underestimation of sepsis severity and lack of adequate resuscitation. Finally, the group non-survivors had a significantly lower Hb, even after correction for the age difference. Theoretically, this could be explained partially by differences in pre-hospital fluid administration, since this parameter was not recorded in the present study. From previous studies, however [11], we know that administration of 2 l normal saline in 1 h results in only a 7.5% decrease in Hb and Ht. So, it is highly unlikely that pre-hospital fluid administration alone accounts for the reported difference in Hb. Since oxygen delivery is partially dependent on available Hb, the lower the Hb, the earlier relative tissue hypoxia will occur, especially in a hypermetabolic state such as sepsis. This finding supports the recommendation of administration of blood in sepsis patients with a ScvO_2_ of less than 70% when other resuscitative methods have already been applied [4].

In our study, the mean time to antibiotics was 87 min, which is less than Barochia et al. [12] reported in a recent meta-analysis investigating the effect of the introduction of “sepsis bundles.” Fluid resuscitation in our study was comparable to what De Miguel-Yanes et al. [13] recently reported in an observational study of patients with uncomplicated sepsis at the ED.

Our findings are of high clinical relevance, since recently Glickman et al. demonstrated that ine out of five patients visiting the ED with uncomplicated sepsis progress to severe sepsis or even septic shock within 72 h [14]. Early identification of risk factors creates an opportunity to tailor individual treatment in patients presenting with uncomplicated sepsis in order to prevent disease progression.

### Limitations

Since the prospective quality improvement initiative to improve early sepsis recognition in patients with sepsis at the ED ran only 3 months, only 70 subjects were included, and a total of 8 events were recorded. This relatively small sample size of our study precludes drawing solid conclusions regarding causality: Except for beta-blocker use, the study had insufficient power to detect univariate correlations of outcome with age, underlying predisposing medical condition, MEDS score, Hb and other clinical and biochemical parameters. Future studies with larger sample sizes should therefore be carried out to address this. As with any retrospective study, the data included and their accuracy are dependent on the original ED documentation. Since no formal data collection protocol was used, important data regarding treatment characteristics of our patient population (e.g., time to antibiotics and the amount of fluid and oxygen administration) were unavailable or incomplete for a significant number of patients at the moment of chart review.

## Conclusions

Non-survivors of uncomplicated sepsis had on average a higher abbreviated MEDS score, a lower Hb and more often used beta-blockers compared to survivors. Early identification of the presence of these factors might contribute to optimization of sepsis treatment for this patient category and thereby prevent disease progression to severe sepsis or septic shock.

## Abbreviations

MEDS score: Mortality in Emergency Department Sepsis score; ED: Emergency department; SIRS: Systemic inflammatory response syndrome.

## Competing interests

The authors declare that they have no competing interests.

## Authors’ contributions

EtA designed the study; EtA, MdJ and IB were involved with data collection. EtA was primary author. GW and JtM were secondary authors and editors of the manuscript. All authors read and approved the final manuscript.
